# Unlocking Blood Drivers of Neurodegeneration: Mechanisms and therapies

**DOI:** 10.1002/alz.083655

**Published:** 2025-01-03

**Authors:** Katerina Akassoglou

**Affiliations:** ^1^ Gladstone Institutes, UCSF, San Francisco, CA USA

## Abstract

**Background:**

Cerebrovascular alterations and innate immune activation are key features of Alzheimer’s disease (AD). However, the mechanisms that link blood‐brain barrier disruption to neurodegeneration are poorly understood and well‐defined druggable targets at the neurovascular interface are limited.

**Method:**

By developing a multiomic and genetic loss‐of‐function pipeline, we reported the transcriptomic and global phosphoproteomic landscape of blood‐induced microglia activation and the causal role for fibrin in induction of neurodegenerative genes and oxidative stress pathways in innate immune cells^1^. Volume imaging in cleared mouse and human AD brains combined with repetitive in vivo two‐photon imaging showed focal fibrinogen deposits associated with loss of dendritic spines independent of amyloid plaques. We developed a first‐in‐class fibrin‐targeting immunotherapy to selectively inhibit fibrin‐induced inflammation without interfering with its beneficial coagulation effects^2^.

**Result:**

We identified the blood coagulation factor fibrinogen as necessary and sufficient for the induction of pathogenic neuroinflammation in neurologic diseases^3^. Fibrinogen induces spine elimination and promotes cognitive deficits in AD mouse models mediated by oxidative stress induction in microglia^4^. Fibrin‐targeting immunotherapy entered the CNS, bound to fibrin, and inhibited amyloid‐driven neurotoxicity and neurotoxic inflammatory gene programs in AD mice^2^.

**Conclusion:**

Thus, fibrinogen links cerebrovascular damage with immune‐mediated neurodegeneration and fibrin therapeutics may have important therapeutic implications inhibiting vascular‐driven neurodegeneration in AD and related conditions.

**References**

1. Mendiola et al. **Nat Immunol** 2023, 24:1173‐1187

2. Ryu et al. **Nat Immunol** 2018, 19:1212‐1223

3. Petersen et al., **Nat Rev Neurosci**. 2018, 19:283‐301

4. Merlini et al. **Neuron** 2019, 101:1099‐1108

